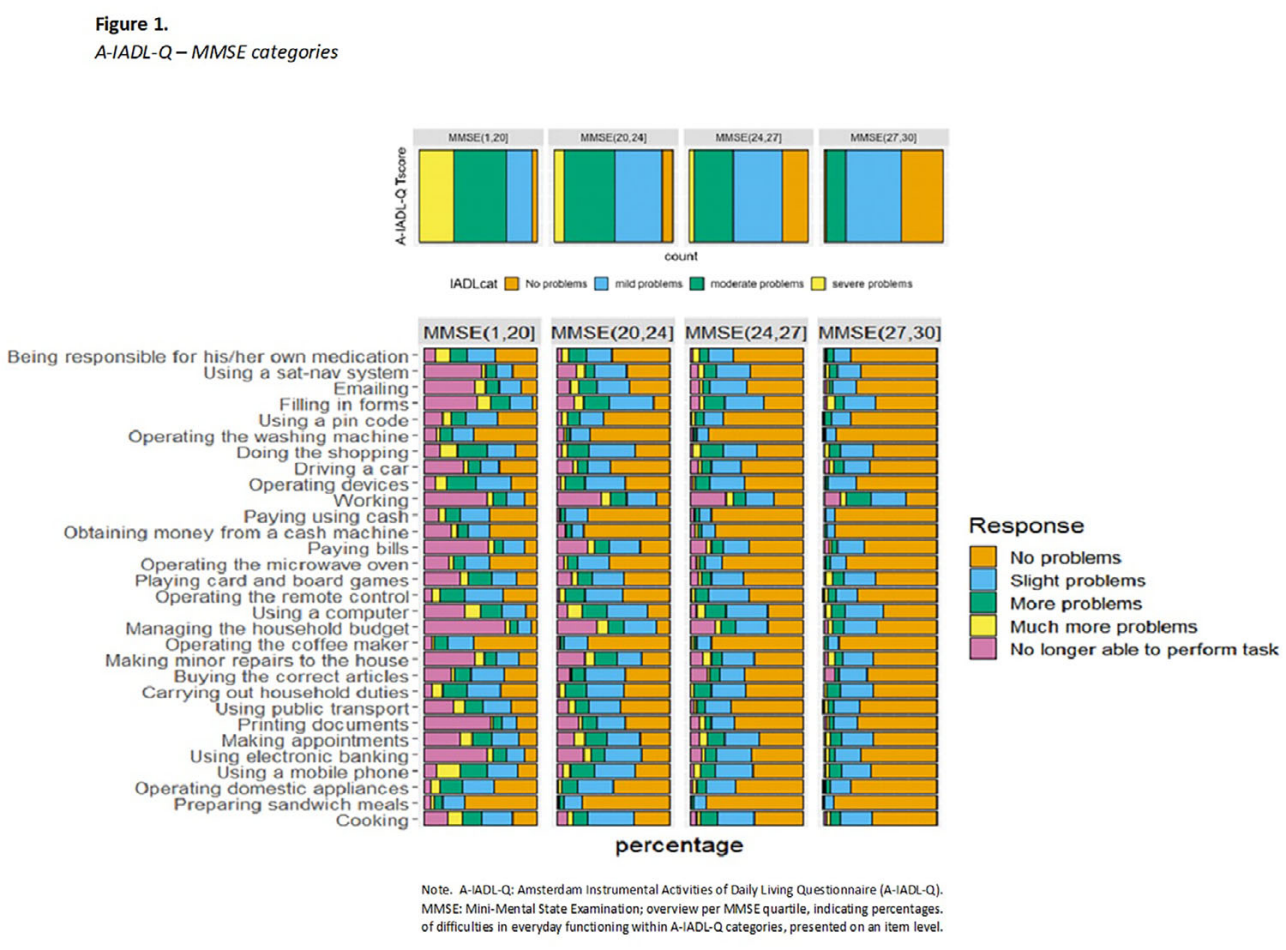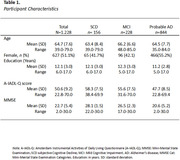# Bridging the Gap between Cognition and Everyday Functioning: A linking Study in Amyloid Positive Participants

**DOI:** 10.1002/alz.092165

**Published:** 2025-01-03

**Authors:** Angela van der Putten‐Toorenburg, Elke Butterbrod, Merel C. Postema, Pieter J. van der Veere, Mukrabe E. Tewolde, Benjamin D. Schalet, Argonde C. van Harten, Elsmarieke van de Giessen, Charlotte Teunissen, Wiesje M. van der Flier, Sietske A.M Sikkes

**Affiliations:** ^1^ Faculty of Behavioural and Movement Sciences, Vrije Universiteit Amsterdam, Amsterdam Netherlands; ^2^ Alzheimer Center Amsterdam, Neurology, Vrije Universiteit Amsterdam, Amsterdam UMC location VUmc, Amsterdam Netherlands; ^3^ Department of Epidemiology and Data Science, Vrije Universiteit Amsterdam, Amsterdam UMC, Amsterdam Netherlands; ^4^ Department of Radiology and Nuclear Medicine, Amsterdam Neuroscience, Vrije Universiteit Amsterdam, Amsterdam UMC, Amsterdam Netherlands; ^5^ Neurochemistry Laboratory, Department of Clinical Chemistry, Vrije Universiteit Amsterdam, Amsterdam UMC location VUmc, Amsterdam, North Holland Netherlands

## Abstract

**Background:**

It remains unclear to what extent global cognition translates to everyday functioning, although this is essential to interpreting the clinical meaningfulness of cognitive deficits. Here, we investigate potential linking between the Mini‐Mental State Examination (MMSE) and the proxy‐based Amsterdam Instrumental Activities of Daily Living Questionnaire (A‐IADL‐Q).

**Methods:**

Cross‐sectional data from 1228 amyloid‐positive participants (age = 64±7yrs; 51.1%F), with Subjective Cognitive Decline(*n* = 156), Mild Cognitive Impairment(*n* = 228) or Probable Alzheimer’s Disease(*n* = 844) were included from the Amsterdam Dementia Cohort. Amyloid positivity was based on amyloid PET or cerebrospinal fluid, according to local cut‐off values. All participants completed MMSE, and all proxies A‐IADL‐Q. Equipercentile linking, unidimensional Item response theory (IRT) calibration and multiple regression analyses were explored as linking methods. For each MMSE quartile, we explored the proportion of reported problems on the A‐IADL‐Q total score and each A‐IADL‐Q item. We described the most commonly affected activities.

**Results:**

Assumptions for direct linking (equipercentile/IRT) were not met (r = 0.53,95%CI[0.48,0.57]). We proceeded with multiple regression analyses, with MMSE as independent variable and A‐IADL‐Q as dependent variable (adjusted for age, sex and education). Higher MMSE scores were associated with higher everyday functioning in the total sample (B = .96;95%CI[0.87,1.04]), with a one‐point increase in MMSE corresponding to nearly one additional point in A‐IADL‐Q scores. In the highest MMSE quartile (27‐30), 35% of participants experienced ‘no problems’, while 47%,17%, and 1% experienced 'mild,’ ‘moderate,’ and ‘severe problems’ respectively (Figure 1). In the lowest quartile, 5% reported 'no problems‘, with 22%,44%, and 29% experiencing 'mild,’ ‘moderate,’ and 'severe problems’. Most commonly affected activities in the highest MMSE quartile were working (74% at least mild problems), followed by using a computer (52%) and managing the household budget (47%). The latter two activities were also most commonly affected in the lowest MMSE quartile, but to a much higher degree (93%,95%) alongside filling in forms (96%).

**Conclusion:**

Although direct linking between cognition and daily functioning was not possible, our findings support more functional dependencies with increasing cognitive problems. Functional difficulties in the highest MMSE group suggest limited sensitivity of cognitive screening. Our findings suggest a nuanced link between global cognition and everyday functioning, supporting complementary use of both instruments.